# Comparative Experimental Assessment of Elastomeric and Thermoplastic Sealing Materials in Valve Sealing Under Cyclic High-Pressure Hydrogen Exposure

**DOI:** 10.3390/polym18070814

**Published:** 2026-03-27

**Authors:** Enric Palau Forte, Francesc Medina Cabello

**Affiliations:** 1 Redfluid, 08227 Terrassa, Spain; 2 Chemical Engineering Department, Universitat Rovira I Virgili, 43007 Tarragona, Spain; francesc.medina@urv.cat

**Keywords:** hydrogen, high-pressure ball valve, sealing materials, ethylene propylene diene monomer rubber, nitrile butadiene rubber, polyether ether ketone, polyoxymethylene

## Abstract

Hydrogen is increasingly adopted as a clean energy carrier for storing and transporting low-carbon energy. Achieving a practical volumetric energy density for real-world deployment typically requires compression to several hundred bar, which in turn demands dedicated high-pressure infrastructure. Because valves are indispensable for isolation and flow control within this infrastructure, durable sealing valve materials become a key reliability and safety requirement. This assembly-level screening study compares two valve configurations with different polymer assemblies: EPDM O-rings with PEEK seats/bushing and NBR O-rings with POM seats/bushing. Four new identical 500-bar ball valves were tested (two EPDM/PEEK and two NBR/POM). For each seal configuration, one valve was cycled 5000 times at 500 bar in helium (inert baseline), and a second identical valve was cycled 5000 times at 500 bar in hydrogen to isolate hydrogen effects from mechanical/metallic wear. Leakage was tracked during cycling, and seals were analyzed by SEM/EDX after testing. The EPDM/PEEK configuration remained leak-tight in both gases, with no cracking observed in the elastomer or thermoplastic components. The NBR/POM configuration exhibited POM bushing fracture during cycling and minor external leakage at the stem during the hydrogen phase, accompanied by micro-fissures on the NBR O-ring surface. EDX indicated composition changes after cycling, including oxygen and fluorine enrichment and occasional metallic transfer species, consistent with surface films and deposits. Under the present valve geometry and cycling protocol, EPDM/PEEK provided robust sealing, whereas NBR/POM showed failure modes relevant to high-pressure service. These findings are intended as configuration-level screening evidence to be used in valves rather than as a full qualification of the individual materials.

## 1. Introduction

Hydrogen is emerging as a key alternative energy source, recognized for its efficiency across diverse applications. However, developing the necessary infrastructure to safely handle hydrogen, particularly under high-pressure conditions, presents significant technical challenges. Among the critical components in this infrastructure are ball valves, which must endure the stresses of high-pressure hydrogen cycles, including frequent opening and closing under fluctuating conditions.

High-pressure compressed hydrogen is one of the most efficient methods for storing large quantities of hydrogen within limited spaces. To ensure the safety and reliability of such infrastructures, valves capable of withstanding extreme pressures are essential components.

**Figure 1:**
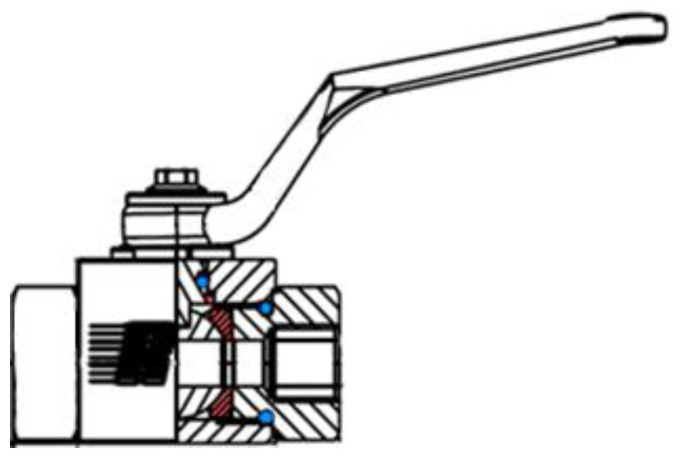
Cross-sectional schematic of a 500-bar ball valve showing the seat/stem-bushing (red) components and O-ring (blue) sealing locations.

A ball valve is composed of various metallic and polymeric components, each serving a specific function to ensure proper sealing, flow control, and durability in demanding environments. While the metallic parts provide the structural integrity, the polymeric components play a crucial role in sealing and wear resistance. Among these, O-rings and gaskets are essential for static and dynamic sealing applications. On the other hand, valve seats and packing are two other critical polymer-based elements that require a different material selection approach. Valve seats act as the sealing interface between the valve closure element (e.g., ball) and the valve body, ensuring the valve shut-off. These components must withstand high mechanical loads, pressure variations, and continuous contact with aggressive media without deformation or excessive wear. The main sealing locations and the seat/stem-bushing arrangement of the studied 500-bar ball valve are shown in Figure 1.

Common elastomers used for O-rings include nitrile rubber (NBR) and ethylene–propylene–diene monomer rubber (EPDM), while typical thermoplastic seat materials include polyoxymethylene (POM, acetal) and polyether ether ketone (PEEK). Each material presents advantages and drawbacks.

Polymeric materials are widely used in hydrogen infrastructure, where elastomeric seals (e.g., O-rings) are commonly employed for both static and dynamic sealing at critical interfaces. Under high-pressure hydrogen exposure, polymeric components may exhibit changes in permeability and dimensional stability, while hydrogen ingress can promote micro-bubble formation and localized damage. During rapid depressurization, the release of trapped gas can result in severe physical degradation, often described as rapid gas decompression (RGD), which represents a major reliability concern for polymer-based components in hydrogen service. Thermoplastics like POM and PEEK within hydrogen exposure—particularly under elevated pressures and repeated high-pressure cycling—can lead to deterioration of key mechanical properties, and endurance shall be assessed. Elastomers such as EPDM and NBR tend to undergo pronounced mass uptake, surface blistering, and hardness loss under sustained hydrogen environments [1], which may progressively impair sealing integrity and increase the leakage risk, with their performance to be reviewed under the most realistic circumstances.

NBR is a widely used industrial sealing elastomer, but its performance in high-pressure hydrogen could be doubtful. In the studies of Castagnet et al. [2] and Qiao et al. [3], the compatibility of NBR with hydrogen is discussed, and it is suggested that NBR is compatible with hydrogen to a certain extent, but its performance is significantly affected by hydrogen permeation and decompression.

EPDM, an unsaturated rubber, is known for excellent weather and ozone resistance, and has shown good stability even after aging in hydrogen at moderate pressures [4]. However, EPDM’s hydrogen permeability is relatively high, which could potentially allow more gas penetration (and leakage) through the seal [4]. Under ageing in a hydrogen environment, EPDM showed the least degradation when compared to HNBR or FKM but higher permeability [4]. Mengru Fang et al. reported, when studying hydrogen permeation under a representative condition (2 MPa, 273 K), permeability reductions of ~83–86% relative to NBR and EPDM. Although obtained from atomistic modeling, these results provide mechanistic support for elastomer-dependent permeability rankings and reinforce that diffusion-controlled hydrogen ingress can differ markedly among elastomer families, motivating comparative seal testing under relevant pressure cycling [5]. In the comparative study by Yijie et al., quantitative comparisons of carbon black-filled EPDM, FKM and HNBR exposed to very high-pressure hydrogen reported superior dimensional stability for EPDM. EPDM surface morphology observations indicated no evident fractures compared with micro-cracking in FKM and HNBR [6].

Polyether ether ketone (PEEK) and polyoxymethylene (POM) are two thermoplastic polymers commonly utilized for ball valve seats. PEEK performs well at elevated temperatures and provides superior mechanical strength, while POM is valued for its excellent dimensional stability and low friction. Although POM is less suitable at high temperatures, it is widely used in valve configurations because of its lower cost compared with PEEK, making it a viable choice when temperature tolerance is less critical.

Polyoxymethylene (POM) is widely used in valve seats where PTFE is not suitable due to PTFE deformation on high pressures, but its susceptibility to brittle fracture under high stress can be a limitation in contrast to PEEK. On the other hand, PEEK is a high-performance thermoplastic with outstanding mechanical strength and thermal stability.

POM’s high crystallinity and chemical inertness suggest moderate compatibility with hydrogen, but its susceptibility to swelling, brittle fracture, and tribochemical wear warrants caution [7]. In contrast, PEEK is a high-performance thermoplastic with outstanding mechanical strength, thermal stability, and chemical inertness; it exhibits much lower gas permeation rates than many other polymers and maintains its properties under high-pressure hydrogen exposure [8]. Recent experimental studies evaluating thermoplastic polymers under high-pressure cyclic hydrogen exposure [9] have provided further insight into the suitability of PEEK and POM for hydrogen service. Under cycling pressures representative of hydrogen infrastructure and temperatures ranging from ambient down to −40 °C, PEEK exhibited negligible changes in mechanical properties, glass transition temperature, crystallinity, and storage modulus, indicating a high resistance to hydrogen-induced physical effects. In contrast, POM showed measurable increases in elastic modulus after hydrogen cycling, attributed to physical interactions between hydrogen and the amorphous regions of the polymer, although no chemical degradation or macroscopic damage was observed. These results suggest that while POM may remain suitable for hydrogen applications under controlled conditions, PEEK offers superior stability and reliability under cyclic high-pressure hydrogen environments [9].

Although specimen-level studies provide valuable insight into the intrinsic response of thermoplastics under high-pressure hydrogen, Zhao et al. [10] highlight that many commonly used ex situ protocols necessarily involve decompression when specimens are removed from a pressure vessel, which can modify free volume and dimensions and therefore bias-derived transport or property metrics; they consequently emphasize the value of in situ evaluation under representative high-pressure H_2_ cycling. Accordingly, valve polymeric seals should be validated under cyclic high-pressure hydrogen in realistic component geometries (e.g., valve seats and bushings), where mechanical constraint, contact stresses, and actuation-driven wear can couple with hydrogen uptake and ultimately govern durability and sealing performance.

Accordingly, the novelty of the present work lies in the comparative assembly-level characterization of commercially relevant sealing configurations in a real valve geometry. This approach complements specimen-level and model-based studies by capturing contact stress, confinement, tribology, actuation and leakage response in the complete component.

Recent work on high-pressure hydrogen valve safety highlights that minor external leakages can initiate through very small, irregular gaps created by damage of the internal sealing ring. Gao et al. investigated seal damage and leakage initiation in high-pressure hydrogen valves under extreme ambient temperatures, reporting that sealing rings in contact with hydrogen are commonly manufactured from EPDM and hydrogenated nitrile butadiene rubber (HNBR). These observations reinforce the need for assembly-level validation of sealing material choices [11].

Another recent computer simulation study on pipeline ball valves found that hydrogen-blended natural gas can make the internal flow more unstable and reduce the pressure that squeezes the seat against the ball during closing, increasing the risk of sealing failure. The same study suggests that a “slow-then-fast” closing motion can help preserve both sealing and flow performance [12]. Nevertheless, there is limited experimental evidence on how seal materials perform in full valve assemblies under repeated high-pressure actuation in hydrogen. This work compares four 500-bar ball valve assemblies with identical geometry but different polymeric seal configurations (EPDM/PEEK and NBR/POM). The assemblies were tested under helium and hydrogen to distinguish changes primarily associated with cyclic actuation under pressure from those associated with hydrogen exposure. Complementary studies from Europe, Korea, and the United States further indicate that hydrogen-related permeability and degradation issues are of broad international relevance [4,8].

By combining leakage monitoring with post-test SEM/EDX characterization, this study aims to provide assembly-level characterization data and engineering evidence for material selection in high-pressure hydrogen valves and related equipment.

## 2. Materials and Methods

### 2.1. Valve Assemblies and Materials

**Scheme 1:**
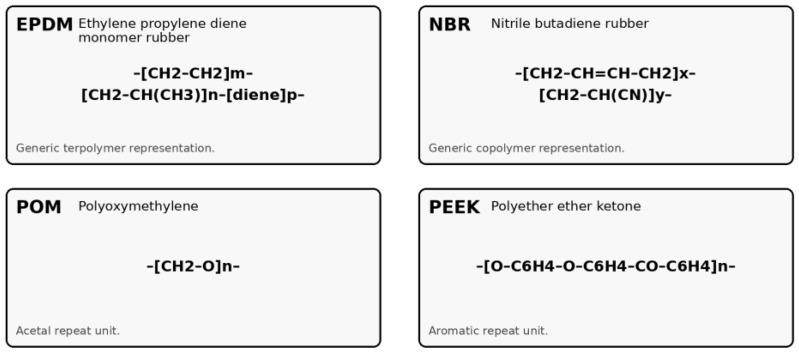
Generic repeat-unit representations of EPDM, nitrile butadiene rubber, polyoxymethylene and polyether ether ketone used in the two valve configurations.

Four high-pressure, two-way ball valve assemblies were prepared, identical in design except for the sealing materials. The ball valves had a nominal diameter of 13 mm, a pressure rating of 500 bar, and 316 L stainless steel for all metallic parts. Each valve incorporates two main polymeric sealing elements critical for tightness: (1) O-rings providing static seals (in the valve body and stem interface), and (2) annular valve seats that seal against the ball to prevent flow when closed and a stem bushing. Two valves were assembled with EPDM O-rings and PEEK seats/stem bushing, and two valves with NBR O-rings and POM seats/stem bushing. Within each configuration, one valve was assigned to helium testing and the other to hydrogen testing (i.e., no valve hardware was reused between gases). These material pairs represent a durable/high-performance combination (EPDM–PEEK) versus a more conventional combination (NBR–POM) whose limits in hydrogen service are evaluated. Generic repeat-unit representations of the investigated polymer families are shown in Scheme 1.

Elastomers (O-rings): The EPDM (ethylene–propylene–diene, peroxide-cured) O-rings and NBR (nitrile rubber, acrylonitrile–butadiene) O-rings were both of an identical size (seal diameter and cross-section) appropriate for the valve design (approximately 8.8 mm ID, 1.9 mm cross-section). Both had hardness in the 70–80 Shore A range, typical for valve seals. The O-rings were new, and lightly lubricated during assembly with a fluorinated grease for aggressive media; therefore, residual lubricant can contribute to the fluorine signal in near-surface EDX measurements.

In each valve, the O-ring’s function was to seal the interface between the two-part valve body (preventing external leakage at the body joint) and to seal around the stem.

Thermoplastics (seats and bushing): The valve seats are ring-shaped inserts that sit between the metal ball and body, providing a soft sealing surface. The seat dimensions and interference were identical for both materials to minimize geometric contributions when comparing seat integrity and leakage behavior.

### 2.2. Test Procedure and Cycle Definition

**Scheme 2:**
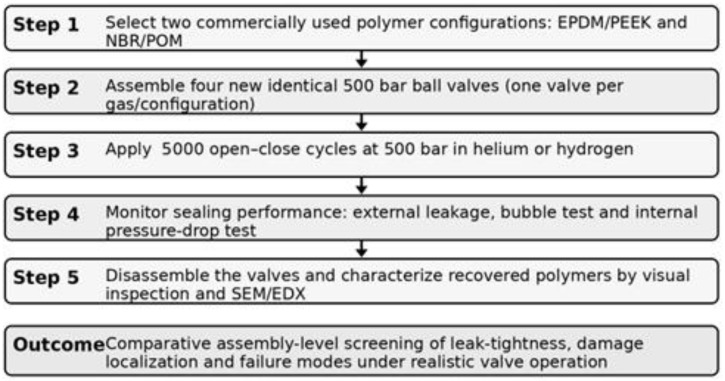
Schematic workflow of the comparative assembly-level evaluation performed on the two sealing configurations under helium and hydrogen cycling.

**Figure 2:**
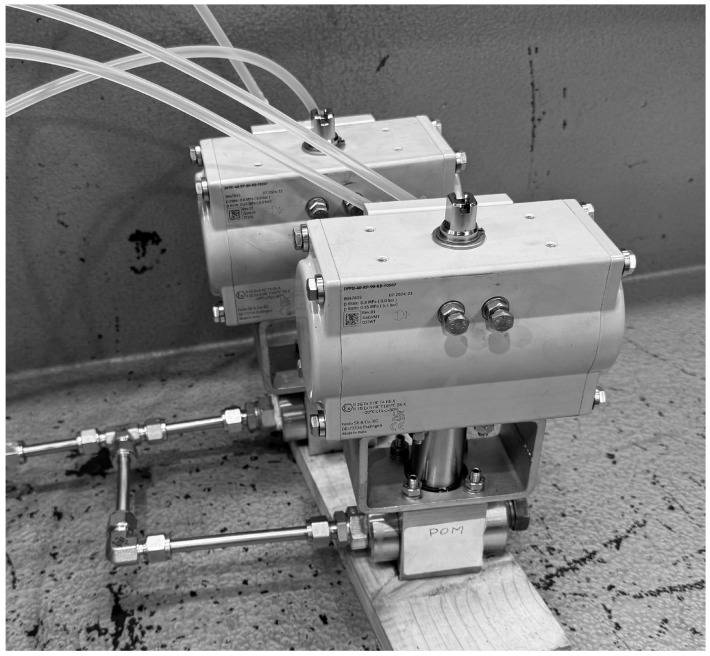
Representative high-pressure ball valve test rig.

The valves were installed in a custom high-pressure cycling test rig. Each valve was connected to a gas supply system capable of pressurizing the inlet side up to 500 bar with either helium or hydrogen. A cycle was defined as one mechanical actuation from fully closed to fully open and back to fully closed, performed while the inlet pressure was regulated at 500 bar. The actuation period was fixed at 3 s per open and 3 s per close cycle. Although this study did not implement a dedicated rapid gas decompression (RGD) qualification protocol, the seal response of EPDM and NBR in high-pressure hydrogen is not governed solely by “RGD events”. In situ measurements under high-pressure hydrogen show that swelling during and after depressurization is strongly controlled by hydrogen transport (especially diffusivity) and by formulation/stiffness effects (e.g., filler reinforcement), with diffusivity correlating more strongly with swelling than equilibrium hydrogen uptake [13]. Therefore, cyclic high-pressure hydrogen exposure tests can provide meaningful insight into seal integrity and leakage performance even when a formal RGD procedure is not included, because the actuation cycles resemble real valve operation. In practice, depressurization to ambient pressure was performed only at the end of each gas-specific test run prior to disassembly and inspection. The overall workflow and the main elements of the 500-bar test rig are summarized in Figure 2. The comparative assembly-level testing workflow used in this study is illustrated in Scheme 2.

Each valve was subjected to 5000 open–close cycles at 500 bar in its assigned gas (helium or hydrogen). Helium tests provided an inert baseline for mechanically driven wear, while hydrogen tests were performed on separate, new valves of the same model to avoid confounding effects from cumulative metallic wear. Depressurization to ambient pressure was performed only at the end of each test run prior to disassembly and inspection. The hydrogen used was 99.999% purity (industrial-grade compressed H_2_). All tests were conducted at ambient temperature (~20–25 °C).

### 2.3. Leak Detection and Monitoring

Throughout cycling, external leakage was assessed at scheduled checkpoints.

External leakage (to atmosphere at the stem/body interfaces) was assessed in the sniffer mode using a mass-spectrometric leak detector (ASM 340 from Pfeiffer, Asslar, Germany).

After each test run, a bubble test was also performed at full test pressure using a laser bubble counter (10 min observation window). “Leak-tight” was defined as (i) zero bubbles detected during the 10 min bubble-counter test, and (ii) a sniffer reading ≤ 50 ppmv at the stem and body sealing locations (tightness criterion adopted from ISO 15848-2:2015 [14]).

Internal leakage (across the closed ball/seat) was evaluated by pressurizing the inlet side, and the valve in closed position, and monitoring pressure drop during 10 min (criterion: ΔP ≤ 1% bar over 10 min).

### 2.4. Post-Test Inspection and Analysis

After each test run (helium or hydrogen), the polymer seals were removed for examination. Visual inspection documented macroscopic damage (cracks, distortions, extrusion). The sealing surfaces were examined by scanning electron microscopy ESEM Quanta 600 (FEI Company, Hillsboro, OR, USA)to identify wear features and micro-cracks. Energy-dispersive X-ray spectroscopy (EDX) was used to quantify near-surface elemental composition at multiple locations on each sample (new state and post-test). Changes in oxygen and fluorine were interpreted as indicators of surface modification and/or deposited films; oxygen enrichment can reflect oxidation and/or surface adsorbates, while fluorine can originate from the fluorinated assembly lubricant or transferred fluorinated films. Trace metallic elements were monitored as potential transfer species from valve hardware.

For each material/condition, multiple EDX spot analyses were acquired on different zones. Table 1 reports one representative spectrum per condition for readability, whereas Table A1 summarizes the full multi-location C, O and F dataset as *n*, mean ± SD and range.

Standardized Fourier-transform infrared spectroscopy and post-cycling mechanical tests were not performed on the recovered components because the present work was designed as full-valve screening and only geometry-specific, limited recovered material was available after disassembly. Therefore, this study does not claim bulk chemical degradation or bulk property retention from the present experiments.

Representative NBR SEM fields were additionally post-processed using a simple normalized edge density descriptor to complement the qualitative surface-damage observations.

Because elastomer and thermoplastic seat materials were varied simultaneously between the two assemblies, the primary comparison is at the level of seal configuration (EPDM/PEEK versus NBR/POM). Component-level SEM/EDX observations and the observed leakage/failure locations are used to contextualize the assembly-level outcomes. The conclusions are therefore intentionally restricted to configuration-level performance under the tested valve geometry and cycling protocol.

## 3. Results

### 3.1. Materials Baseline Analysis

At baseline (new, as-installed state), EDX showed carbon-rich surfaces for all polymers (Table 1). NBR exhibited the highest carbon fraction (88.9 wt%) with oxygen at 6.8 wt% and fluorine at 0.7 wt%. EPDM showed 77.8 wt% C, 10.2 wt% O and 3.8 wt% F. POM showed 70.8 wt% C, 9.9 wt% O and 17.7 wt% F, while PEEK showed 80.4 wt% C, 17.3 wt% O and 1.2 wt% F. Because a fluorinated lubricant was used during assembly, fluorine detected on nominally fluorine-free polymers should be interpreted as a near-surface condition rather than bulk composition. Because the cycled surfaces were spatially heterogeneous, the EDX values are reported as representative near-surface values from multiple analyzed locations and are discussed descriptively.

In summary, prior to any gas exposure, all materials were dominated by carbon; EPDM and PEEK contained some oxygen, POM uniquely had a considerable fluorine presence, and NBR had negligible amounts of elements other than carbon.

### 3.2. Helium Pressure Cycling Performance

**Figure 3:**
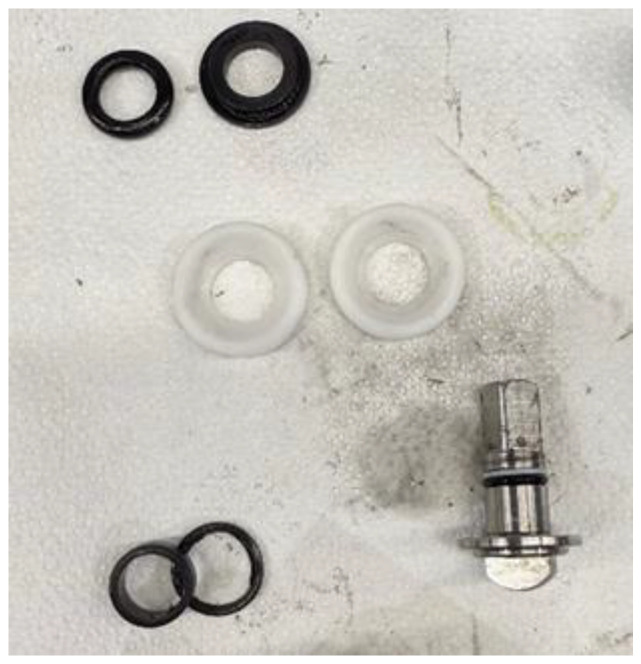
Brittle fracture of POM bushing after high-pressure helium cycling. The image shows a POM valve bushing that cracked into two pieces. No similar failure was observed in PEEK seats.

During the helium cycling phase, the EPDM/PEEK valve remained leak-tight through the target 5000 cycles at 500 bar. No internal or external leakage was detected during periodic leak checks, meeting the strict no-bubble criterion. The brittle fracture observed in the POM bushing after helium cycling is shown in Figure 3.

Visual inspection after helium testing revealed the PEEK seats to be intact with no cracks or deformation, and the EPDM O-rings appeared unchanged (still elastic, with no visible damage).

In the NBR/POM valve, increased actuation torque and an audible event were observed during cycling (≈1500 cycles). Post-test disassembly revealed fracture of the POM bushing. The NBR O-rings did not show gross failure in helium. After helium cycling, a small external leak in the stem region was detected during post-test checks, while internal seat leakage was not detected. A large radial crack had propagated through the POM bushing. The NBR O-ring in that assembly remained unbroken. The POM thermoplastic could not withstand the cyclic compressive and tensile stresses at 500 bar, even in an inert environment (helium). The failure was brittle in nature, with the fracture surface of POM appearing crystalline and without significant plastic yielding.

In contrast, the PEEK seats in the EPDM/PEEK assembly showed no visible cracking or structural damage after an identical helium cycling regime—the valve retained leak-tight closure throughout the helium test (no detectable internal or external leakage).

Following helium cycling, all materials exhibited altered surface elemental profiles compared to their baseline state. NBR remained largely carbon-based (~78.7% C) but showed the incorporation of oxygen (~11%) and an increase in fluorine (~4.6%) relative to the baseline NBR (which had virtually no O and only ~0.7% F). EPDM underwent a more pronounced shift under helium: its carbon content dropped from ~77–78% to around ~50%, while oxygen rose to ~23% and fluorine to ~16%. Furthermore, trace amounts of inorganic elements (not present initially) were detected on the helium-exposed EPDM surface, notably magnesium, silicon, and iron.

For the thermoplastics, POM after helium cycling showed ~80 wt% C, ~10 wt% O, and ~8.8 wt% F. This reflects a slight increase in the carbon fraction and roughly a halving of the fluorine content compared to baseline POM (~17% F initially), with oxygen remaining virtually unchanged (around 9–10%).

Helium-cycled PEEK showed a significant reduction in carbon content (down to ~54–66%) accompanied by an increase in oxygen (~25%), along with the emergence of fluorine at ~11–14% (where F was nearly undetectable in new PEEK). In addition, small quantities of magnesium and silicon were found on the surface of PEEK after helium exposure.

Overall, helium cycling resulted in oxygen and fluorine enrichment on multiple polymer surfaces, consistent with surface modification and/or deposited films generated during high-pressure actuation.

### 3.3. Hydrogen Pressure Cycling Performance

During hydrogen cycling, the EPDM/PEEK valve tested in hydrogen remained leak-tight (no increase above background in sniffing measurements) through the target 5000 cycles at 500 bar. The assembly passed internal and external leak checks as described in the methodology, and post-test SEM inspection did not reveal cracking of the EPDM O-rings or PEEK sealing components.

**Figure 4:**
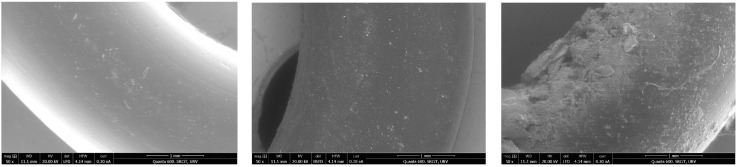
From (**left**) to (**right**), SEM of NBR O-ring surface at baseline and after He and H2 cycles. NBR shows small fissures after H2 cycling test.

**Figure 5:**
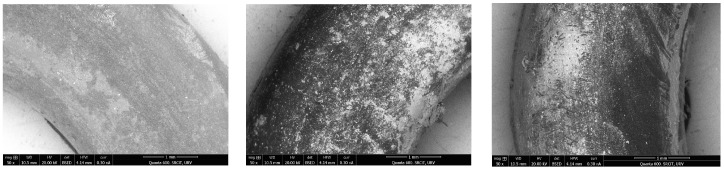
From (**left**) to (**right**), SEM of EPDM O-ring surface at baseline and after He and H2 cycles. The EPDM rubber shows an intact, crack-free surface. The texture is smooth apart from some adhered debris (bright spots) and slight abrasion.

**Figure 6:**
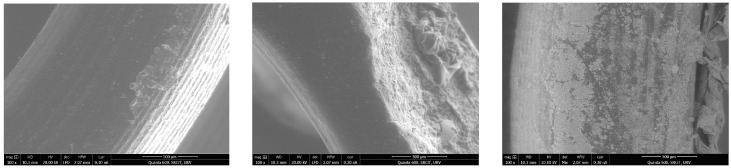
From (**left**) to (**right**), SEM of POM surface at baseline and after He and H2 cycles.

In the NBR/POM valve, a hairline crack was observed on the POM bushing after completing the 5000-cycle test. At around ≈3100 cycles, an increase in actuation torque appeared. Minor external leakage in the stem region was detected toward the end of the hydrogen phase in the bubble leak test. SEM examination revealed fine surface fissures on the NBR O-ring, whereas EPDM surfaces remained comparatively smooth with only minor surface impressions and embedded particulates. As a simple image-based quantification of the NBR surface condition, a normalized edge-density descriptor calculated from representative SEM fields increased from 1.00 in the new state to 1.62 after He cycling and 3.41 after H2 cycling, consistent with the greater fissuring observed after hydrogen exposure. For clarity, the SEM panels in Figure 4 and Figure 5 are representative 50× fields, and the scale bar in each panel corresponds to 1 mm, whereas Figure 6 is 100 fields and 500 μm.

Hydrogen exposure led to more pronounced surface chemistry changes than helium, as evidenced by EDX analysis. Hydrogen exposure caused even more drastic compositional changes, characterized by a substantial decrease in carbon content and a marked increase in fluorine on all polymer surfaces. NBR after hydrogen exposure contained only about ~43% C (down from ~89% in the unexposed material), indicating a significant loss of carbon relative proportion. Concurrently, oxygen in NBR rose to ~22%, and fluorine climbed to ~22–24%—a dramatic enrichment from the mere ~0.7% F at baseline. EPDM likewise retained only ~50% C after hydrogen exposure and showed a major fluorine increase, with fluorine levels reaching ~35%. Additionally, new metallic elements (including chromium, manganese, nickel, and iron) were detected in certain regions of the hydrogen-exposed EPDM surface. These observations are consistent with heterogeneous surface films and deposits formed during cycling

For the thermoplastics after hydrogen cycling, the POM carbon content fell to ~41% (from ~71% initially), while oxygen rose substantially to ~26% (compared to ~9% at baseline). The fluorine content in hydrogen-aged POM was measured in the range of ~18–29%, which is on par with or slightly above its original ~17% F level. In PEEK, hydrogen exposure led to the most extreme changes among all the materials: the carbon fraction dropped to roughly ~28–39%, and, strikingly, the fluorine content skyrocketed up to ~61% in some areas (from virtually 0% in pristine PEEK). Additional EDX spots on PEEK indicated strong spatial variability, with fluorine reaching >60 wt% at some locations. Across all materials, EDX indicates substantial changes in the near-surface elemental balance after cycling, particularly oxygen and fluorine enrichment, which should be interpreted as surface-state modification and/or deposited films rather than direct evidence of bulk chemical reactions.

Overall, under cyclic high-pressure exposure in full valve assemblies, the EPDM/PEEK configuration maintained leak-tight performance in helium and hydrogen, whereas the NBR/POM configuration exhibited POM bushing fracture and minor stem leakage during hydrogen cycling. EPDM maintained seal integrity without cracks, whereas NBR suffered micro-cracking. The EDX results demonstrate significant near-surface composition changes after cycling; however, EDX does not detect hydrogen and cannot uniquely distinguish oxidation from other forms of oxygen- and fluorine-containing surface deposits.

## 4. Discussion

This work provides an assembly-level comparison of two polymer sealing configurations in 500-bar ball valve assemblies subjected to repeated open–close actuation and tested separately in helium and hydrogen (one new valve per gas per configuration). Because elastomer and seat/bushing materials were varied simultaneously (EPDM/PEEK versus NBR/POM), the primary outcomes are configuration-level leak-tightness and damage localization. Post-test SEM/EDX supports the interpretation of component degradation but does not constitute a fully factorial separation of elastomer and thermoplastic effects.

### 4.1. Elastomer Seals: EPDM vs. NBR

In hydrogen cycling, the EPDM/PEEK configuration remained leak-tight and did not show elastomer cracking, whereas the NBR/POM configuration developed minor stem leakage and NBR surface fissures. Under the present actuation protocol, these fissures are plausibly associated with the combined effects of mechanical cycling under high pressure and hydrogen exposure.

Hydrogen transport properties provide useful context for the different damage responses observed in EPDM and NBR seals. Using ex situ desorption-based methods after 24 h H_2_ charging in the 2–11 MPa range, Jung et al. reported comparable hydrogen solubility for carbon-black-filled EPDM and NBR, but a markedly higher diffusivity for EPDM (≈25–27 × 10^−11^ m^2^ s^−1^) than for NBR (≈9–11 × 10^−11^ m^2^ s^−1^), resulting in higher overall permeability for EPDM (P_EPDM > P_NBR). These data highlight an engineering trade-off: EPDM may enable faster through-seal H_2_ transport (potentially increasing steady-state permeation), while the lower diffusivity of NBR can promote longer hydrogen retention within the elastomer during cyclic loading and pressure transients. In the present 500-bar valve-assembly cycling tests, NBR O-rings exhibited fine surface fissures whereas EPDM remained crack-free, indicating that permeability alone does not predict sealing durability under cyclic high-pressure hydrogen service and that material selection should jointly consider leakage control and resistance to damage [15].

Prior studies on rubbers exposed to high-pressure hydrogen have also reported material-dependent differences between EPDM and NBR in permeation and post-exposure behavior. These divergent outcomes are consistent with literature reports that EPDM is intrinsically more resistant to hydrogen-induced damage than NBR [16]. In prior work at 35–70 MPa hydrogen, NBR O-rings absorbed the most hydrogen (~950 ppm) but showed a propensity for crack formation under rapid decompression, whereas EPDM exhibited no cracking at similar pressures [16]. O-ring leakage behavior under hydrogen permeation conditions has been reported [17] to be highly sensitive to assembly parameters that govern interfacial contact stress. When combining experiments and simulations, the leakage rate was reported to decrease first and then increase with pre-tightening torque, while increasing with O-ring inner diameter, indicating a trade-off between improved initial sealing and increased stress that may reduce service life. The same study [17] reported decreasing leakage with an increasing hydrogen pressure and hydrogen blending ratio, attributed to pressure-induced enhancement of O-ring contact stress. These results support the interpretation that configuration-level leakage outcomes in assembled valves are influenced not only by material compatibility but also by the effective sealing stress state established by geometry and preloading, motivating explicit control/reporting of torque or pre-compression in future factorial qualification campaigns.

EDX indicated oxygen and fluorine enrichment on both EPDM and NBR after cycling (Table 1), together with occasional metallic transfer species detected on EPDM in localized areas. Because fluorine was introduced via a fluorinated assembly lubricant, fluorine-rich surface layers cannot be uniquely assigned to bulk polymer chemistry. Similarly, oxygen enrichment can reflect oxidation and/or adsorbed or deposited species, and it may also be influenced by post-test air exposure. Therefore, EDX results should be interpreted as describing a near-surface state modified by tribological processes and deposits, rather than direct evidence of hydrogen-specific chemical attack.

### 4.2. Thermoplastic Seats: PEEK vs. POM

A similarly clear contrast was observed for the seat/bushing components at the assembly level. PEEK seats and bushings remained intact with no detectable leakage under either gas, whereas POM bushings exhibited brittle cracking/fracture. The occurrence of POM fracture already during helium cycling indicates a predominantly mechanical failure mode under the imposed stresses, while the additional stem leakage observed during hydrogen cycling suggests a reduced sealing margin for the NBR/POM configuration under the intended service gas.

EDX showed substantial fluorine on POM surfaces already in the new state and after cycling, consistent with retained lubricant residues. The presence of fluorine did not prevent mechanical fracture of POM. In PEEK, fluorine enrichment after cycling indicates the accumulation of fluorinated films in the near-surface region; spot analyses revealed strong spatial variability, consistent with heterogeneous deposition during high-pressure actuation.

From an engineering perspective, these results support the use of high-performance polymer combinations (e.g., EPDM/PEEK) for high-pressure hydrogen valve service when leak-tightness and mechanical integrity under repeated actuation are critical. The NBR/POM configuration exhibited failure modes (bushing fracture and stem leakage) that reduce reliability at 500 bar. For clarity, the configuration-level outcomes are summarized in Table 2.

Limitations:

- Cross-combinations (e.g., EPDM/POM or NBR/PEEK) were not evaluated.
- EDX characterizes the near-surface region and is sensitive to transferred films and post-test exposure, so it does not uniquely identify bulk chemical degradation mechanisms.
- Only one valve per gas per configuration was tested; therefore, the results should be interpreted as configuration-level screening evidence.

## 5. Conclusions

1. Under 500-bar open–close cycling (5000 cycles per gas), the EPDM/PEEK configuration remained leak-tight in both helium and hydrogen tests (performed on separate valves), with no cracking observed in the elastomer or thermoplastic components.

2. In the NBR/POM configuration, POM bushing fracture occurred in helium testing and minor stem leakage plus NBR surface micro-fissures were observed in hydrogen testing.

3. Helium versus hydrogen exposure: POM fracture during helium cycling indicates a mechanical limitation for POM bushings in this valve geometry under the imposed stresses. Hydrogen cycling was associated with additional leakage in the NBR/POM configuration and stronger near-surface composition changes in multiple materials.

4. Near-surface composition changes: EDX showed oxygen and fluorine enrichment after cycling and occasional metallic transfer species on selected surfaces. Because a fluorinated lubricant was used during assembly, fluorine-rich surface layers are consistent with deposited films/residues, and their origin cannot be uniquely assigned from EDX alone. Oxygen enrichment cannot be uniquely attributed to oxidation. EDX does not detect hydrogen and therefore does not establish hydrogen-specific chemical reactions.

5. Engineering implication: For high-pressure hydrogen ball valves subjected to repeated actuation at 500 bar, the EPDM/PEEK configuration provided a larger sealing margin than the NBR/POM configuration under the conditions tested. These findings should be interpreted as screening guidance for further qualifications under the tested conditions.

## 6. Patents

- Testing device for evaluation of potential fugitive emissions (Spanish patent application). Application number: P202530350; filing date: 25 April 2025, 13:20 (CEST); current status: pending; applicant: Redfluid SL; inventors: Enric Palau Forte; Francesc Medina Cabello.
- Test bench for detecting fluid leaks (Spanish patent application). Application number: P202530237; filing date: 20 March 2025, 15:37 (CET); current status: pending; applicant: Redfluid SL; inventors: Enric Palau Forte; Francesc Medina Cabello.

## Figures and Tables

**Table 1:** Representative EDX near-surface composition of the polymer materials (wt%) in new state and after helium and hydrogen cycling. Values are representative multi-location surface analyses and are interpreted descriptively because the post-cycling surfaces were heterogeneous.

Material	Condition	C (wt%)	O (wt%)	F (wt%)
EPDM	New	77.82	10.22	3.80
EPDM	He	50.11	23.46	15.97
EPDM	H_2_	50.55	9.10	34.80
NBR	New	88.94	6.75	0.73
NBR	He	78.69	11.18	4.57
NBR	H_2_	42.99	21.96	21.76
PEEK	New	80.38	17.28	1.18
PEEK	He	53.74	25.43	10.76
PEEK	H_2_	39.01	13.43	42.56
POM	New	70.77	9.88	17.67
POM	He	79.96	9.76	8.83
POM	H_2_	41.28	26.42	18.74

**Table 2:** Comparative experimental results of seal configurations under 500 bar and 5000 open–close cyclic exposure.

O-Ring	Bushing/Seat	He Cycles (5000)	H2 Cycles (5000)	Failure
EPDM	PEEK/PEEK	Leak-tight; no anomalies observed	Leak-tight; no anomalies observed	No cracking; minor surface deposits observed by SEM/EDX
NBR	POM/POM	Increased torque; POM bushing fracture identified post-test	Minor stem leakage; POM bushing crack observed	POM brittle cracking/fracture; NBR surface micro-fissures after H_2_ cycling

## Data Availability

The data presented in this study are available on request from the corresponding author due to industrial copyright.

## Abbreviations

The following abbreviations are used in this manuscript:

H_2_	Hydrogen
SEM	Scanning electron microscopy
EDX (or EDS/EDX)	Energy-dispersive X-ray spectroscopy
POM	Polyoxymethylene
PEEK	Polyether ether ketone
PTFE	Polytetrafluoroethylene
NBR	Nitrile butadiene rubber
EPDM	Ethylene propylene diene monomer rubber
RGD	Rapid gas decompression

## Appendix A. Descriptive Statistics of the Multi-Location EDX Spot Analyses

**Table A1:** Descriptive summary of the raw multi-location EDX spot analyses used to interpret Table 1.

Material/Condition	*n*	C (wt%)	O (wt%)	F (wt%)
EPDM, New	2	77.40 ± 0.59 [76.98–77.82]	8.76 ± 2.07 [7.29–10.22]	6.75 ± 4.17 [3.80–9.70]
EPDM, He	4	42.53 ± 22.96 [22.85–71.63]	23.87 ± 6.89 [14.80–31.35]	19.78 ± 11.47 [7.89–35.23]
EPDM, H_2_	5	43.90 ± 18.64 [25.68–72.60]	8.21 ± 5.94 [3.16–18.06]	24.33 ± 12.75 [12.03–41.03]
NBR, New	6	70.70 ± 14.71 [55.38–88.94]	12.77 ± 7.34 [3.82–21.63]	0.26 ± 0.29 [0.00–0.73]
NBR, He	2	80.25 ± 2.21 [78.69–81.81]	10.57 ± 0.86 [9.97–11.18]	4.11 ± 0.65 [3.65–4.57]
NBR, H_2_	2	41.95 ± 1.46 [40.92–42.99]	21.73 ± 0.32 [21.51–21.96]	23.06 ± 1.84 [21.76–24.36]
PEEK, New	4	78.00 ± 4.28 [71.64–80.69]	14.14 ± 4.96 [8.31–19.10]	0.29 ± 0.59 [0.00–1.18]
PEEK, He	2	59.64 ± 8.34 [53.74–65.53]	21.61 ± 5.41 [17.78–25.43]	12.31 ± 2.20 [10.76–13.87]
PEEK, H_2_	5	39.16 ± 17.49 [24.72–68.66]	12.36 ± 2.62 [9.14–14.84]	36.83 ± 20.56 [15.22–61.37]
POM, New	2	76.19 ± 7.67 [70.77–81.61]	10.19 ± 0.43 [9.88–10.49]	12.13 ± 7.83 [6.59–17.67]
POM, He	3	80.37 ± 4.16 [76.43–84.72]	9.50 ± 2.54 [6.84–11.89]	8.87 ± 2.23 [6.66–11.11]
POM, H_2_	2	41.48 ± 0.29 [41.28–41.69]	23.46 ± 4.19 [20.50–26.42]	23.61 ± 6.89 [18.74–28.49]

Note: Values were calculated from all available spot analyses reported in the raw laboratory EDX output. Blank fluorine entries in the laboratory report were treated as 0 wt% (not detected) for this descriptive summary.
